# Prevalence of prehypertension and associated cardiovascular risk profiles among prediabetic Omani adults

**DOI:** 10.1186/1471-2458-8-108

**Published:** 2008-04-07

**Authors:** Shyam Sundar Ganguly, Mohammed Ali Al-Shafaee, Kamlesh Bhargava, Kalyan Kanti Duttagupta

**Affiliations:** 1 Department of Family Medicine & Public Health, College of Medicine & Health Sciences, Sultan Qaboos University, Muscat, Oman

## Abstract

**Background:**

The importance of prediabetes and prehypertension has been demonstrated in several studies especially for primary prevention of CVD. A recent community based cross-sectional study revealed that 40.9 percent Omani adults are prediabetics. This study was undertaken to estimate the prevalence of prehypertension and associated cardiovascular risk profiles in prediabetics. To best of our knowledge, this is the first report on this subject.

**Methods:**

The study included 327 confirmed pre-diabetic Omani adults, who were analysed for their demographic, metabolic and behavioral characteristics. These characteristics were compared between the three different blood pressure groups to determine the CVD risk factors. Univariate and step-wise multiple logistic regression methods were used to carry out the analysis of the data.

**Results:**

In this study, the prevalence of prehypertension was 54.1 percent. Males were at higher risk of developing prehypertension as compared to females (OR = 2.30, 95% CI: 1.21, 4.38; P < 0.01). The individuals with higher BMI have two fold more risk of developing prehypertension (OR = 2.25, 95% CI: 1.26, 4.02; P < 0.01). The increased level of OGT enhanced the risk of developing prehypertension (OR = 1.26, 95% CI: 1.06, 1.50; P < 0.01).

**Conclusion:**

A high prevalence of prehypertension (54.1%) exists in this study population. The major determinants of prehypertension in these prediabetic subjects were male gender, increasing dysglycemia and BMI. Appropriate intervention strategies have been suggested.

## Background

Sultanate of Oman has been successful in reducing communicable diseases and increasing life expectancy at birth to 76 years for females and 71 years for males. Rapid cultural changes and social advances since 1970 have led to the manifestation of wide range of non-communicable diseases [1]. A high prevalence of diabetes (11.6%), obesity (20.5%), hypertension (27.0%) and metabolic syndrome (21.0%) exists in Omani population particularly in urban dweller and older individuals [1-4]. Obesity is recognized as independent risk factor for hypertension, diabetes mellitus and dyslipidaemia, which are also known to be independent risk factors for cardiovascular diseases (CVD) [5,6].

It has been documented that the patients with diabetes have twice the risk of incidence of myocardial infarction and stroke as that of the general population. As many as 80 percent of patients with type 2 diabetes mellitus will develop and possibly die of macro vascular diseases. This represents a great societal cost with major loss of life expectancy and quality of life [7]. Recent studies have demonstrated that individuals in prediabatic state can be identified and diabetes can be delayed if not prevented [8]. Similarly, the prehypertension category is designed to focus physicians, patients and public attention on blood pressure (BP) in the 120–139 mm Hg systolic and/or 80–89 mm Hg diastolic range with the aim to slow down or prevent the progression of rising blood pressure, arterial stiffness and kidney damage that occurs over time [9].

In a recent community-based cross-sectional study, carried out in a semi-urban locality in Oman, revealed that 40.9% of the study subjects were prediabatics. In this study, all the non-diabetic Omani adults (18 years and above) residents of Wilayat (district) of Bidbid were screened for prediabetes based on impaired fasting glucose (IFG) and oral glucose tolerance test (OGTT). The study included impaired glucose tolerance (IGT; 2_h glucose 7.8 – 11.0 mmol/l), impaired fasting glucose (IFG; fasting glucose 5.6–6.9 mmol/l) as per American Diabetes Association (ADA) criteria [10].

The strong Heart Study reported that the prevalence of prehypertension among diabetics to be 59.4% as compared to 48.2% in non-diabetic participants. Impaired fasting glucose and impaired glucose tolerance also greatly increased cardiovascular disease risk in prehypertensive adults without apparent association with incident cardiovascular disease among normotensive participants [11]. In San Antonia Heart Study, 1440 prehypertensive subjects were followed up for 8 years. Hypertension developed in 130 subjects (9.03%). Clustering of atherogenic changes associated with hypertension actually preceded the development of hypertension [12]. Moreover, the adverse consequences of prehypertension were increased in the presence of diabetes mellitus and hypercholesterolemia [13].

Prehypertension is a risk factor for overt hypertension, and several small-scale studies have demonstrated its association with increased CVD morbidity. Prehypertension doubles the risk of future CVD events [14]. The risk of CVD in individuals with prehypertension has not been thoroughly studied in prediabetic adults. We herein report the prevalence of prehypertension among prediabetic Omani adult population and evaluate the relationship between the CVD risk profiles and blood pressure status of the subjects. This will be helpful to the health planners for formulation and implementation of preventive strategies.

## Methods

### Study population

Three hundred twenty seven confirmed prediabetic Omani adults (142 males and 185 females) above 18 years of age, who participated in a cross-sectional community based study carried out in a semi-urban locality of Bidbid Wilayat (district), situated about 100 km south of the capital Muscat during September 2004 to February 2005 formed the material of this study.

Fasting blood glucose, fasting lipid profile, oral glucose tolerance tests were carried out. The anthropometric, systolic and diastolic blood pressure measurements were also carried out. The Medical Research and Ethics Committee of the College of Medicine and Health Sciences, Sultan Qaboos University approved the study and all subjects gave their informed consent prior to participating in the study.

Brachial artery BP (first and fifth Korotkoff sounds) was measured 3 times consecutively on seated participants after they had rested 5 minutes with the use of a standardized mercury sphygmomanometer. An appropriately sized cuff was placed on the right arm, pulse occlusion pressure was determined, and the cuff was inflated to 20 mm Hg above that pressure. The mean of the last two of these measurements was used for estimation of BP [15]. The standard definition of hypertension was considered as systolic blood pressure (SBP) of ≥ 140 mm Hg and/or diastolic blood pressure (DBP) ≥ 90 mm Hg or current use of antihypertensive medicines. Prehypertension was defined as SBP of 120 – 139 mm Hg and/or a DBP of 80–89 mm Hg. The normotensive was defined as SBP < 120 mm Hg and DBP < 80 mm Hg [8].

The fasting plasma glucose (FPG), Oral glucose tolerance test (OGTT) levels and the lipid profile were estimated at the Sultan Qaboos University Hospital (SQUH) according to standard protocol.

We defined high total cholesterol (TC) as ≥ 5.0 mmol/l; triglycerides (TG) as ≥ 1.7 mmol/l; LDL cholesterol (LDL-C) as ≥ 3.0 mmol/l; HDL cholesterol (HDL-C) as ≤ 1.03 mmol/l. The body mass index (BMI) was calculated as weight/height^2 ^(kg/m^2^), and over weight was defined by considering the cutpoint as BMI ≥ 25 kg/m^2^.

Waist circumference was measured using steel measuring tape with measurements made half way between the lower border of the ribs and iliac crest in a horizontal plane. Hip circumference was measured at the widest point over the buttock. Waist hip ratio (WHR) was considered abnormal ≥ 0.95 for males and ≥ 0.85 for females [16].

Individuals who reported a history of diabetes diagnosed by a physician and who were taking any medicine or insulin for the disease and those who were not on medicine but their fasting plasma glucose found to be > 7.0 mmol/l were excluded from the study. Women found pregnant during the survey period were also excluded from the study.

### Statistical analysis

Descriptive analysis for continuous variables was used to calculate mean values and standard deviation (SD). Prevalence and frequencies were expressed as percentages. Chi-square tests were used for establishing relationship between the two categorical variables. Chi-square trend test was used for trend analysis of proportions. Unpaired t tests were used for comparing two group means and analysis of variance (ANOVA) techniques were used for comparing three group means. When ANOVA provided the evidence of significant difference between the three categories then post hoc test namely, Dunnett t test was used for pairwise group means comparison. The unadjusted odds ratios (ORs) and their 95% confidence intervals (CI) were calculated for association between the risk profile categories and hypertensive status of the subjects.

To evaluate the adjusted effects of CVD risk profiles in the prehypertensive group as compared to normotensive group, multiple logistic regression models were fitted and OR with their 95% CI were estimated. The risk profile variables entered in the regression model were age, gender, BMI, WHR and lipid parameters namely, TC, LDL-C, HDL-C and TG. For estimating the multivariate adjusted ORs corresponding to the potential risk factors, we first fitted the model after including all the risk factor variables and compared with the adjusted ORs obtained after refitting the model using stepwise regression procedure [17]. A p-value (two tailed) of less than 0.05 was considered as statistical significant. All data were analyzed with the Statistical Package for the Social Sciences Software (SPSS version 10.0, Inc., Chicago, IL)

## Results

Characteristics and lipid profiles of the study population is shown in Table 1. It is observed that the mean age of the female prediabetics was more than that of males (P < 0.05). The mean values of the oral glucose tolerance tests (OGTT), BMI and HDL-C were more in females (p < 0.01). The WHR, SBP, DBP and TG have been found to be more in males and the differences were statistically significant (p < 0.05). Further, the analysis of blood pressure of the study population showed that 54.1% were prehypertensives and 24.2% were hypertensives.

**Table 1:** Characteristics and lipid profiles of the prediabetic study group

Characteristics	Total (n = 327)	Male (n = 142)	Female (n = 185)	p
			
	Mean ± SD	Mean ± SD	Mean ± SD	
Age (years)	35.0 ± 12.0	33.4 ± 12.3	36.2 ± 12.0	0.039
FPG (mmol/L)	5.9 ± 0.4	5.9 ± 0.4	5.8 ± 0.4	0.059
OGT (mmol/L)	7.1 ± 1.8	6.6 ± 1.7	7.6 ± 1.7	<0.001
SBP (mmol/L)	125.7 ± 15.9	128.6 ± 16.4	123.4 ± 15.2	0.003
DBP (mmol/L)	78.9 ± 9.9	80.3 ± 10.0	77.8 ± 9.8	0.027
BMI (kg/m^2^)	27.1 ± 5.6	26.2 ± 4.7	27.9 ± 6.2	0.004
WHR	0.89 ± 0.08	0.90 ± 0.07	0.88 ± 0.09	0.014
TC (mmol/L)	5.0 ± 1.1	5.0 ± 1.0	5.1 ± 1.1	0.492
LDL-C (mmol/L)	3.4 ± 0.9	3.4 ± 0.9	3.4 ± 0.9	0.595
HDL-C (mmol/L)	1.2 ± 0.3	1.1 ± 0.3	1.3 ± 0.3	<0.001
TG (mmol/L)	0.9 ± 0.6	1.0 ± 0.7	0.8 ± 0.5	<0.001
Smoking history (%)	14.1%	18.3%	10.8%	0.040
BP status (%)				
Normotensive	21.7%	16.2%	25.9%	0.024
Prehypertensive	54.1%	53.5%	54.6%	
Hypertensive	24.2%	30.3%	19.5%	

The demographic, metabolic and behavioral characteristics of the study population as per blood pressure groups are shown in Table 2. It is observed that except for HDL-C and TG, all other biochemical parameters have shown a gradual upward trend from normotensive group to prehypertensive and further to hypertensive group. As the blood pressure increased from normotension to hypertension group, the average age of the study population showed an increasing trend. Prehypertensive category had significantly higher level of TG and BMI in comparison to normotensive category (p < 0.01). Similarly, hypertensive subjects had higher level of FPG and LDL-C in comparison to prehypertensive. The proportion of male subjects increased linearly from 32.4% in normotensive to 42.9% in prehypertensive and further to 54.4% in hypertensive group (p < 0.01).

**Table 2:** Demographic, Metabolic and Behavioral characteristics of prediabetic subjects by BP groups (Mean ± SD)

Variable	Normotensive (n = 71)	Prehypertensive (n = 177)	p*	Hypertensive (n = 79)	p^↑^
Age (years)	33.1 ± 9.8	33.2 ± 11.0	0.979	40.7 ± 14.7	<0.001
Sex, male/female	23/48	76/101	0.064	43/36	0.044
FPG, (mmol/L)	5.8 ± 0.4	5.9 ± 0.4	0.263	6.0 ± 0.4	0.050
IGT (mmol/L)	6.7 ± 1.7	7.2 ± 1.8	0.062	7.4 ± 1.8	0.567
TC (mmol/L)	4.8 ± 0.9	5.0 ± 1.0	0.238	5.3 ± 1.2	0.076
LDL-C (mmol/L)	3.3 ± 0.8	3.4 ± 0.9	0.412	3.6 ± 1.0	0.050
HDL-C (mmol/L)	1.2 ± 0.3	1.2 ± 0.3	0.756	1.2 ± 0.4	0.972
TG (mmol/L)	0.7 ± 0.5	0.9 ± 0.6	0.011	0.9 ± 0.6	0.771
BMI (kg/m^2^)	25.2 ± 4.6	27.6 ± 5.8	0.002	27.7 ± 5.8	0.899
WHR	0.87 ± 0.09	0.89 ± 0.07	0.740	0.92 ± 0.08	0.010
Smoking history %	19.7%	13.0%	0.179	11.4%	0.720

* p-values for comparison between normotensive and prehypertensive subjects

^↑ ^p-values for comparison between prehypertensive and hypertensive subjects

The sexwise distribution of metabolic parameters in different BP groups is shown in Table 3.

**Table 3:** Mean age, metabolic parameters and BMI by BP groups and sex

Variable	Normotensive (n = 71)			Prehypertensive (n = 177)			Hypertensive (n = 79)		
			
	Males (n = 23)	Females (n = 48)		Males (n = 76)	Females (n = 101)		Males (n = 43)	Females (n = 36)	
			
	Mean ± SD	Mean ± SD	P	Mean ± SD	Mean ± SD	p	Mean ± SD	Mean ± SD	p
Age (years)	32.3 ± 10.3	33.5 ± 9.7	0.634	30.9 ± 10.2	34.9 ± 11.3	0.018	38.3 ± 15.1	43.5 ± 14.0	0.123
FPG (mmol/L)	5.8 ± 0.3	5.8 ± 0.4	0.402	5.9 ± 0.4	5.8 ± 0.4	0.321	6.0 ± 0.4	6.0 ± 0.4	0.464
IGT (mmol/L)	5.9 ± 1.5	7.1 ± 1.7	0.005	6.6 ± 1.7	7.7 ± 1.7	<0.001	6.9 ± 1.9	7.9 ± 1.6	0.011
TC (mmol/L)	4.6 ± 0.9	4.9 ± 0.9	0.248	5.0 ± 0.9	5.0 ± 1.1	0.951	5.1 ± 1.2	5.4 ± 1.2	0.213
LDL-C (mmol/L)	3.1 ± 0.8	3.3 ± 0.8	0.400	3.4 ± 0.8	3.4 ± 1.0	0.835	3.5 ± 1.1	3.8 ± 1.0	0.183
HDL-C (mmol/L)	1.1 ± 0.2	1.3 ± 0.3	0.005	1.1 ± 0.3	1.3 ± 0.3	<0.001	1.2 ± 0.4	1.2 ± 0.3	0.878
TG (mmol/L)	0.8 ± 0.4	0.6 ± 0.5	0.101	1.1 ± 0.8	0.7 ± 0.5	<0.001	0.9 ± 0.6	1.0 ± 0.6	0.633
BMI, (kg/m^2^)	25.5 ± 3.9	25.0 ± 4.9	0.703	26.2 ± 4.9	28.7 ± 6.2	0.004	26.4 ± 4.8	29.4 ± 6.6	0.022
WHR	0.91 ± 0.07	0.86 ± 0.10	0.049	0.90 ± 0.08	0.88 ± 0.08	0.133	0.92 ± 0.07	0.92 ± 0.09	0.835

It is seen that OGT is higher in females than males amongst all the three BP groups and the sex differential in each category were found to be statistically significant (p < 0.01). HDL-C level in women was significantly higher in normotensive and prehypertensive as compared to males (p < 0.05). The BMI was found to be significantly higher in females in both prehypertension and hypertension groups as compared to males (p < 0.05).

Sex wise prevalence of risk factors for CVD and estimation of odds ratios are shown in Table 4. It is found that the BMI was the strongest modifiable predictor of prehypertension and hypertension in females with OR 4.0 (95% CI, 1.8, 8.8) and OR 4.0 (95% CI, 1.4, 11.3) respectively. The TC and LDL-C showed an upward trend in both sexes amongst the prehypertensive group as compared to normotensive group. The prediabetic male individuals with lower level of HDL-C (< 1.03 mmol/l) had two fold risk of being classified as prehypertensive as compared to normotensives. Females with abnormal WHR were found more at risk for developing hypertension with OR 2.7 (95% CI, 0.9, 8.1).

**Table 4:** Prevalence of risk factors (%) for CVD by BP groups and gender in the prediabetic subjects

Variable	Males (n = 142)						Females (n = 185)				
			
	Normal (n = 23)	Pre-HTN (n = 76)	OR (95%CI)	HTN (n = 43)	OR (95%CI)		Normal (n = 48)	Pre-HTN (n = 100)	OR (95%CI)	HTN (n = 36)	OR (95%CI)

TC (≥5.0 mmol/L)	39.1	51.3	1.6 (0.6, 4.7)	48.8	1.5 (0.5,4.7)		43.8	47.0	1.2 (0.6, 2.4)	63.9	2.3 (0.9, 6.1)
TG (≥ 1.7 mmol/L)	-	23.7	-	11.6	-		4.2	5.1	1.2 (0.2,9.5)	11.1	2.9 (0.4,24.3)
LDL-C (≥ 3.0 mmol/L)	59.1	71.1	1.7 (0.6,5.1)	73.7	1.9 (0.6,6.9)		66.7	71.3	1.3 (0.6,2.8)	75.0	1.5 (0.5,4.4)
HDL-C (≤ 1.03 mmol/L)	34.8	52.6	2.1 (0.7, 6.2)	32.6	0.9 (0.3,3.0)		18.8	18.0	1.0 (0.4,2.5)	25.0	1.5 (0.5,4.6)
BMI (≥25.0 kg/m^2^)	60.9	59.2	0.9 (0.3,2.7)	53.5	0.7 (0.2,2.3)		39.6	72.3	4.0 (1.8,8.8)	72.2	4.0 (1.4,11.3)
Abnormal WHR (%)	17.4	31.7	2.2 (0.6, 8.5)	34.9	2.5 (0.6, 10.7)		56.3	65.3	1.5 (0.7, 3.1)	77.8	2.7 (0.9, 8.1)
Smoking history (%)	26.1	18.4	0.6 (0.2,2.2)	14.0	0.5 (0.1,1.9)		16.7	8.9	0.5 (0.2,1.5)	8.3	0.5 (0.1,2.1)

The determinants of prehypertension versus normotension based on fitting multiple logistic regression models in prediabetic subjects are shown in Table 5. The stepwise logistic regression method revealed that the males were at higher risk of developing prehypertension as compared to females with OR 2.30 (95% CI, 1.21, 4.38; p < 0.01). The individuals with higher BMI have two fold more risk of developing prehypertension with OR 2.25 (95% CI, 1.26, 4.02; p < 0.01). The analysis also revealed that one unit increase in the OGT level increases the risk of developing prehypertension with OR 1.26 (95% CI, 1.06, 1.50; p < 0.01) in prediabetics.

**Table 5:** Determinants of prehypertension versus normotension based on fitting multiple logistic regression models in the prediabetic subjects

Variable	Adjusted ORs after including all the factors in the model				Adjusted ORs after eliminating the insignificant factors using stepwise regression		
			
	OR	95% CI	P		OR	95% CI	P

Male sex	2.41	1.11, 5.21	0.026		2.30	1.21, 4.38	0.011
Blood glucose (IGT mmol/l)	1.28	1.06, 1.53	0.009		1.26	1.06, 1.50	0.009
BMI (≥25 kg/m^2^)	1.87	1.00, 3.50	0.050		2.25	1.26, 4.02	0.006

The OR for blood glucose was continuous and calculated for 1 unit.

## Discussion

The overall prevalence of Prehypertension in prediabetic Omani population was estimated to be 54.1 percent. It is marginally higher in females (54.6%) in comparison to males (53.5%) (Table 1). The main determinants of prehypertension were increasing BMI and blood sugar levels. The male prediabetics were more susceptible to prehypertension. Hsai et al in their study found a prevalence of 40.3 percent prehypertensives among Asian women [18]. Epidemiological data from National Health and Nutrition examination survey, USA estimated the prevalence of prehypertension to be 31%. Additionally, more than 88% of the individuals with prehypertension had at least one cardio vascular risk factor [19]. Prevalence estimated in our study is less than the prevalence of pre-hypertension reported in the Strong heart study among diabetics [11]. This indicates that prevalence of prehypertension is more in diabetics than prediabetics.

In a study among young Israeli adult population the prevalence of prehypertension has been estimated to be 50.6% in males and 35.9% in females [14]. Our finding when compared to all other studies on general population indicates that prevalence of prehypertension is more among pre-diabetics for both males and females.

In Framingham Heart Study, 6859 participants, who were initially pre-hypertensive and were followed up for 10 years. The cumulative incidence of CVD in the subjects between 35 – 64 years was 8% in males and 4% in females. In older persons above 65 years, it increased to 25% for males and 18% for females [20].

Winegarden [21] in his study estimated Relative Risk (RR) for an individual becoming a hypertensive from prehypertensive status. He divided prehypertension group into two subcategories namely, normal (120–129 systolic and/or 80–85 diastolic) and high normal (130–139 systolic and/or 86–89 diastolic). He estimated RR for the "normal" subcategory as 2.0 (95% CI, 1.6, 2.6) and for "high normal" as 2.9 (95% CI, 2.3, 3.7).

In a study of cardiovascular risk factor status, 614 non diabetic Mexican American were followed up for 8 years and 43 (7.0%) developed pre-diabetes and had high level of TC, LDL-C, TG, BMI and BP and lower level of HDL-C than 571 subjects remained non-diabetic [22]. Similar finding was also noticed in our study population (Table 1). It is likely that prediabetic subjects have an atherogenic pattern of risk factors and may contribute to the risk of macro vascular disease. It has been estimated that if prehypertension is eliminated, hospital admission would be reduced by 3.4%, nursing home admission by 6.5% and death would be reduced by 9.1% [23].

In our study the prehypertensives were found to have higher level of FPG, OGT, TC, LDL-C, TG and BMI than normotensives (Table 2, 3, 4). Similar findings were reported by Liszka et al [19] and Grotto et al [14]. Haffner et al [12,22] in their two studies reported similar findings amongst both prehypertensives and prediabetics. This clustering of risk factors indicates the need of cardiovascular risk reduction in prehypertensives and prediabetics through primordial prevention. The stepwise multiple logistic regression analysis indicated that the individuals with higher blood sugar, BMI and male sex are at higher risk of developing prehypertension (Table 5). Israeli et al found that the BMI is the strongest modifiable predictor of prehypertension [24].

Prospective observational studies suggest the risk of cardio vascular death begins at 115/75 mm Hg and doubles for every 20/10 mm Hg increment in a linear fashion [9].

Life style measures such as medical nutrition therapy and aerobic exercises modify lipids, reduce blood pressure and are integral to the management of prediabetes and obesity. Patients with prehypertension and prediabetes should initiate life style modifications like weight control, increased physical activity, alcohol moderation, salt reduction and increased consumption of fresh fruits, vegetable and low fat diary product for 3 months [7]. Physical activities should be at least 150 minutes of moderate intensity aerobic physical activities or at least 90 minutes of vigorous exercise per week. If after these efforts, targets are not achieved, treatment with pharmacological agent should be initiated [7]. Tobacco cessation intervention program should be introduced in health centers and hospitals as a routine because it is a priority of state of Art diabetic and hypertension care.

Washio et al [25] in their study estimated that in Japanese population those with prehypertension had an increased risk of coronary atherosclerosis even after adjusting for other factors when compared to subjects with normal blood pressure and concluded that prehypertension is a clinical entity which requires treatment. In Oman, consanguinity is very common. Hassan et al [26] in their study, carried out in an urban sample of Omani population, found that 26% of the marriages were within their first cousin. At the national level this figure is very high. The first cousin marriage under the age of 50 years in 1995 was about 54% of total marriages [26]. This cultural practice may result into increase in both diabetes and hypertension.

## Conclusion

The true question regarding prehypertension amongst prediabetics is the CVD risk associated with this condition and anticipated risk reduction to be gained by early initiation of primordial, primary and early diagnosis and treatment. A long term study will be required to understand the risk associated with prehypertension in prediabetics and diabetics and role of pharmacological agents to bring down the BP to 115/75 mm Hg to reduce CVD in Omani population.

## Competing interests

The author(s) declare that they have no competing interests.

## Authors' contributions

SSG conducted the literature review, statistical analyses, drafted and edited the manuscript. MA-S provided expertise and oversight throughout the process and reviewed the drafts. KB provided expertise and oversight throughout the process. KKD provided expertise, edited sections and reviewed the drafts. All authors read and approved the final manuscript.

## Pre-publication history

The pre-publication history for this paper can be accessed here:


